# Providing Japanese health care information for international visitors: digital animation intervention

**DOI:** 10.1186/s12913-018-3191-x

**Published:** 2018-05-21

**Authors:** Mariko Nishikawa, Masaaki Yamanaka, Junko Kiriya, Masamine Jimba

**Affiliations:** 1 Department of Global Health and Nursing, Graduate School of Nursing, University of Human Environments, Nagoya, 3-220, Ebata cho, Obu city, Aichi 474-0035 Japan; 2 Department of Maritime Science and Technology, Japan Coast Guard Academy, Kure, Japan; 3 Department of Community and Global Health, Graduate School of Medicine, The University of Tokyo, Tokyo, Japan

**Keywords:** Digital animation, Health information, International visitors, Japan

## Abstract

**Background:**

Over 24 million international visitors came to Japan in 2016 and the number is expected to increase. Visitors could be at a risk of illness or injury that may result in hospitalization in Japan. We assessed the effects of a four-minute digital animation titled *Mari Info Japan* on the level of anxiety experienced by international visitors to Japan.

**Methods:**

We conducted a non-randomized, controlled study at Narita International Airport outside Tokyo in December 2014. On the first day, we recruited international visitors for the intervention group at predetermined departure gates and, the following day, we sampled visitors for the control group at the same gates. We repeated this procedure twice over 4 days. The intervention group watched the digital animation and the control group read a standard travel guidebook in English. After receiving either intervention, they completed a questionnaire on their level of anxiety. The outcome was assessed using the *Mari Meter-X*, The State-Trait Anxiety Inventory Form Y (STAI-Y), and a face scale, before and immediately after the intervention. We analyzed data with Wilcoxon rank sum tests.

**Results:**

We recruited 265 international visitors (134 in the intervention group, 131 in the control group), 241 (91%) of whom completed the questionnaire. Most of them had no previous Japanese health information before arrival in Japan. The level of anxiety about health services in Japan was significantly reduced in the intervention group (*Mari Meter-X* median: − 5 and 0, *p* < 0.001 and STAI-Y median: − 3 and 0, *p* < 0.001). The face scale analysis showed no significant difference.

**Conclusions:**

Watching a digital animation is more effective in reducing anxiety among international visitors to Japan compared with reading a standard brochure or guidebook. Such effective animations of health information should be more widely distributed to international visitors.

**Trial registration:**

UMIN-CTR (University Hospital Medical Information Network Center Clinical Trials Registry), UMIN000015023, September 3, 2014.

**Electronic supplementary material:**

The online version of this article (10.1186/s12913-018-3191-x) contains supplementary material, which is available to authorized users.

## Background

The number of international visitors worldwide has grown sharply, from 0.65 billion in 1996 to 1.22 billion in 2014 [1]. In Japan, the number increased from 8.6 million in 2010 to over 24 million in 2016, attracted by a government advertising campaign [2]. Furthermore, this number will rise further as the Olympic games will be held in Tokyo in 2020 [3].

International visitors are at risk of injury or illness in visiting countries. Of the total number of American visitors who returned from Asia and Sub-Saharan Africa, approximately 75% experienced travel-related illnesses due to gastrointestinal, febrile, and dermatologic diseases [4]. Other problems were sun-related conditions, sexually transmitted diseases, insect bites, jellyfish stings, or animal-related injuries [5–7]. In Japan, international visitors went to hospitals with symptoms of diarrhea, abdominal pain, or fever [8, 9]. In extreme cases, they required surgery [10, 11]. Some international visitors eschewed a hospital when ill because of the expense or their unfamiliarity with hospital procedures [12].

As the number of international visitors sharply increases, there will be a greater demand for appropriate knowledge to deal with prevention and treatment. Major international events, such as the Olympic games, require effective health information strategies for international visitors [13–16].

Meanwhile, there are three main problems with health information for international visitors. First, in their home countries, health education for travelers is usually provided through brief notes in brochures, arbitrary and subjective material on the Internet, or in guidebooks [17–19]. Moreover, although the Health Ministries of many countries provide health related information, it is mostly for their own citizens going abroad and the majority of pre-travel health information is about infectious diseases in developing countries [7, 20, 21]. Information on other health related topics for tourists coming from abroad are also required in the host countries.

Second, the effectiveness of pre-travel education depends on the health facility visited [19, 22]. In some countries, primary physicians and nurses may not be up-to-date about travel health issues [23].

Third, succinct travel guidelines are available for Japanese nationals [24], but they are inappropriate for international visitors. For example, international visitors are primarily concerned about paying medical expenses, communication, and informed consent at health facilities, but sufficient information to reduce these concerns are inadequately provided [12].

Host countries have a stake in providing information to international visitors about healthy living and health systems [5], illness prevention [6, 25] and procedures to access health facilities [5, 12, 26]. Travelers have a healthy and enjoyable experience when timely and critical knowledge is available before visiting other countries [27, 28]. Hosts can also use such information when their friends or relatives visit them and ask about health issues [29–31].

**Fig. 1:**
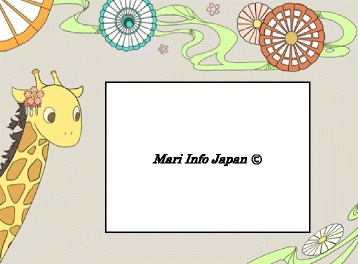
Digital animation: Mari Info Japan


Effective intervention is vital to providing practical information. A digital animation can provide culturally and linguistically appropriate information about preventive health behaviors [32–34]. An earlier study showed that an educational digital video of in vitro fertilization (IVF) was a more effective medium for understanding health risks than a brochure, for couples starting their first cycle of IVF [35]. This tool can be applied to conveying information on travel medicine to international visitors. In this study, we examined the effect of a four-minute digital animation titled *Mari Info Japan* (Fig. 1, Additional files 1 and 2) on the level of anxiety experienced by international visitors to Japan.

**Additional file 2:** *Mari Info Japan* in English. It shows the full version of the digital animation for this study. (WMV 8291 kb)


## Methods

### Study design and procedures

We conducted a non-randomized controlled study on the effectiveness of video instruction among international visitors to Japan on the level of anxiety experienced by them. In this intervention study, we recruited participants at departure gates for the United States, Canada, and the United Kingdom at Narita International Airport, Japan, from December 21 to 24, 2014.

**Fig. 2:**
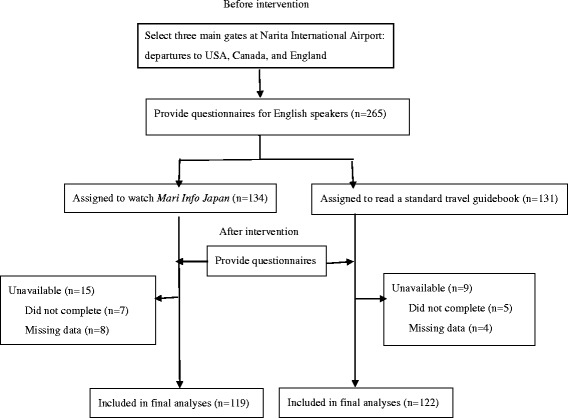
Trial profile

The participants answered a questionnaire about anxiety before receiving either intervention. Thereafter, they answered another questionnaire to evaluate any changes to their anxiety (Fig. 2). We evaluated the differences in anxiety level before and after the intervention, and between the intervention and control groups.

### Participant entry

#### Sample size

We estimated a sample size of 120 visitors in each group to have a 95% confidence level and 80% power to detect a difference of 3.1 in the questionnaire score in our analyses.

#### Eligibility

We recruited international visitors at departure gates to the aforementioned countries, where English is the primary language. They had more time to participate in this study while waiting for their flights than those who had just arrived. In addition, targeting gates for English speaking countries enabled us to find more people eligible for this study. The questionnaire was only in English to avoid any discrepancy in translation. We approached adults who were 20 years old or over, who could read, listen to, and write English. To maximize the validity of responses to our questionnaire, we adhered to a strict criterion. We recruited only those who were calmly seated at their gate at least 2 h prior to departure. This ensured they could concentrate on their responses without being rushed.

#### Enrolment procedure

Since we recruited visitors in our study at a small number of gates, it was difficult to provide both the experimental and control interventions to them at the same time while avoiding contamination. We sampled participants for the intervention group at a particular gate on the first day and recruited the control group the following day. We repeated this procedure twice over 4 days. We invited those who were already at the gates when we started recruitment. Thereafter, we asked every person who arrived at the gates if they would participate in this study. We recruited participants based on their willingness to tell us about their knowledge of the Japanese health system and health information. If they were travelling with friends or family members, we asked them to respond to the questionnaire individually.

### Interventions

We left participants alone in both groups after providing a short explanation on how to view the video (in the intervention group) and answer the questionnaire to avoid any distraction.

#### Intervention procedure

The intervention group watched a four-minute digital animation in English titled *Mari Info Japan* (Fig. 1). This animation aimed to provide information on health care system in Japan for international visitors. Its content is based on a previous study, which found that international visitors’ main concern was how to visit health facilities [12]. It started with traditional Japanese music and contained 11-items (see Fig. 1, Additional files 1 and 2). We provided an 8 × 5-in. *digital notepad* and a headset to each visitor.

#### Control procedure

The control group read a simple information sheet in English, adopted from a standard travel guidebook [24] (see Additional file 3), which was expected to take approximately 3 min to finish reading.

### Outcome assessment

The primary outcome of this study was the level of anxiety the visitors experienced about the Japanese health system and visiting health facilities. We assessed the outcome using STAI-Y, *Mari Meter-X* and a face scale before and after the visitors participated in the interventions.

1. STAI-Y [36] is a 40-item questionnaire, using a four-point Likert-type scale. Twenty questions were about their *state anxiety*, which is the level of anxiety they feel when imagining they became ill. The other 20 questions were about *trait anxiety*. This reveals the level of anxiety that they generally feel in their day-to-day lives. The visitors completed both the state anxiety and trait anxiety questions before the trial. After the trial, the visitors answered only about their state anxiety. The STAI-Y scores are separated into state anxiety (20–80 points) and trait anxiety (20–80 points).
2. *Mari Meter-X* [12, 31] is a 15-item questionnaire using a five-point Likert-type scale (see Additional file 4), which examines the level of anxiety regarding visiting health facilities in Japan. The total score ranged from 15 to 75 points.
3. The face scale [37] measured the visitors’ feelings as indicated by face illustrations on a six-point Likert-type scale. They were asked to choose one face that represented their overall feelings at the time of data collection. It is the easiest and simplest way to express feelings. We assessed feelings the participants expressed simply using this face scale as well as detailed feelings measured with the above-mentioned questionnaires.

We used a demographic questionnaire (Table 1), the *Mari Meter-X*, STAI-Y and face scale. The visitors took about 15 min to complete the questionnaires. While they were watching the digital animation, one of the coauthors was nearby to answer any questions and collect the device after viewing. At no stage, did we hurry, chat with, or interrupt the visitors while they were completing the questionnaire.

**Table 1:** Demographic profile of participants

				*n* = 241
Participant characteristics		Intervention group *n* = 119 *n* (%)	Control group *n* = 122 *n* (%)	*p*
Gender (*n* = 237)	Male	76 (64)	68 (57)	] 0.370
	Female	42 (36)	51 (43)	
Age (*n* = 241)	20<30	69 (59)	54 (44)	] 0.166
	30<40	27 (23)	29 (24)	
	40<50	12 (10)	19 (15)	
	≦50	11 (8)	20 (16)	
Travel with conductor (*n* = 241)	Yes	1 (1)	3 (2)	] 0.325
	No	118 (99)	119 (92)	
Visiting days^b^ (*n* = 241)		10	10	–
Visiting times (*n* = 241)	Once	51 (43)	45 (37)	] 0.329
	More than once	68 (57)	77 (63)	
Visiting medical facility (*n* = 240)	Yes	15 (13)	40 (33)	] < 0.001^***^
	No	104 (87)	81 (67)	
Medical Insurance (*n* = 240)	Yes	102 (86)	104 (86)	] 0.504
	No	17 (14)	17 (14)	
Reason to not have insurance^a^	Did not know	2	5	–
	Have confidence	4	6	–
	Unnecessary expense	10	6	–
	Other	0	3	–
Previous health information (*n* = 241)	Yes	23 (19)	22 (18)	] 0.796
	No	96 (81)	100 (82)	
Source of the information^a^	Brochure	6	9	–
	Web	12	5	–
	Health Facilities from home country	2	3	–
	Colleagues and friends	7	11	–
Regions (*n* = 241)	USA	62 (52)	62 (51)	–
	Canada	9 (7)	10 (8)	–
	China	10 (9)	0 (0)	–
	UK	7 (6)	21 (17)	–
	Indonesia	6 (5)	0 (0)	–
	France	3 (3)	3 (3)	–
	Germany	4 (3)	0 (0)	–
	Australia	3 (3)	3 (3)	–
	New Caledonia	4 (3)	3 (2)	–
	Singapore	2 (2)	3 (2)	–
	Other (11 countries)	9 (7)	17 (14)	–
STAI - Y (trait anxiety score) before the trial^b^ (*n* = 241)		61	61	–

^a^Multiple answers

^b^Median

#### Other information

As this was a non-randomized controlled study, we collected data on the background of the visitors, such as age, sex and previous visit to Japan, their home countries and visits to health facilities during their stay in Japan to determine if the characteristics of the visitors were balanced between the groups, as well as to detect factors that could affect the outcome other than the intervention.

### Bias prevention

We could not ensure that the visitors were blind to their group allocation. However, there was very little chance that the visitors would know if they were either in the intervention or control group as we recruited visitors for each group on different days. Furthermore, the person who analyzed the data was not aware of the participants’ intervention allocation.

#### Data analysis

For the main outcome, we analyzed the difference in median scores for the *Mari Meter-X,* STAI-Y state anxiety, and face scale before and after the intervention for each group with a two-sided Wilcoxon signed rank test. We compared differences in the before and after intervention scores between groups and conducted a multiple regression including all other variables to evaluate the effectiveness of the intervention, adjusted for other potential factors that affect the outcome. We conducted all statistical analyses using the JMP statistical package (version 11.0).

## Results

In our study, 300 international visitors were approached and 265 agreed to participate in the study. We excluded data from 24 visitors (9%) for various reasons: 12 did not complete either the intervention or the questionnaire because they started boarding or fell asleep during the intervention, while 12 questionnaires contained missing data (Fig. 2). We analyzed data from a total of 241 visitors, 119 in the intervention group and 122 in the control group.

### Characteristics of visitors and baseline comparison

Table 1 shows a summary of the basic characteristics of the intervention group (*n* = 119) and control group (*n* = 122). The groups were balanced with respect to baseline characteristics, except for “visiting a medical facility in Japan,” which was greater in the control group (*n* = 40; 33%) than in the intervention group (*n* = 15; 13%) (*p* < 0.001). More than half of the visitors were men in both groups. Over 40 % belonged to the 20–30-year age-bracket in both groups. Both groups had no travel coordinator and stayed in Japan for a median of 10 days. Over half of the visitors came to Japan two or more times in both groups. In total, 86% of the visitors in both groups held medical insurance. The reasons for not possessing travel insurance were viewing it as an unnecessary expense, having confidence in their health status, or not knowing the issues.

For the question on what to do when they get sick or injured during their trip, the most common responses were find a hospital (*n* = 138), look for a pharmacy (*n* = 67), call the consulate for their home country (*n* = 45), ask at hotel reception (*n* = 40), call an ambulance (n = 40), call their tour conductor (*n* = 12), and other (*n* = 36). Both groups had little knowledge of Japanese health information. Among the visitors, 96 (81% of the total) in the intervention group and 100 (82% of the total) in the control group did not obtain health information before coming to Japan. Their sources of information were brochures, websites, hospital or clinic in their home country, colleagues, and friends. Their countries of origin were well balanced between groups: the United States of America, Canada, the United Kingdom, France, and Australia. However, only participants in the intervention group came from China, Germany, Indonesia, New Caledonia, and Singapore.

### Reliability

The high reliability of the *Mari Meter-X* was confirmed by a Cronbach’s alpha internal consistency reliability coefficient of 0.94, and the coefficient for the STAI-Y was 0.92 in this study.

### Statistical analysis

Table 2 shows scores for both groups, before and after intervention, analyzed by Wilcoxon signed rank tests. There was no significant difference in the STAI-Y trait anxiety scores, which evaluated their general feeling before receiving the intervention, between the intervention and control groups. A statistically significant reduction in the *Mari Meter-X* score before and after the intervention was observed in the intervention group (median changed from 46 to 39, *p* < 0.001). Similarly, the reduction in STAI-Y state anxiety scores before and after receiving the intervention was statistically significant (median changed from 50 to 43, *p* < 0.001). However, the difference in the face scale score before and after intervention was not statistically significant.

**Table 2:** Outcomes for before vs. after test for same groups by Wilcoxon signed rank test

	Before				After				*p*-value
		(Median)				(Median)			
	Q1	Q2	Q3	Range	Q1	Q2	Q3	Range	
Intervention group									(*n* = 119)
Mari Meter-X	37	46	55	15–70	29	39	43	15–65	0.001^***^
STAI-Y: state anxiety	38	50	59	30–72	33	43	50	30–69	0.001^***^
Face Scale	0	1	2	0–5	0	1	2	0–4	0.278
Control group									(*n* = 122)
Mari Meter-X	35	46	55	15–75	35	44	55	15–75	0.214
STAI-Y: state anxiety	40	52	58	30–70	39	48	57	30–69	0.007^**^
Face Scale	0	1	2	0–5	0	1	2	0–5	0.577

Q1 The first quartile

Q2 The second quartile

Q3 The third quartile

^**^p < 0.01

^***^p < 0.001

In the control group, the *Mari Meter-X* score did not decrease much from before to after the intervention, whereas the STAI-Y state anxiety score was reduced. The face scale showed no difference before and after the intervention. The *Mari Meter-X* showed that the participants were more likely to be concerned about paying medical expenses (median = 4), language communication (median = 4), and informed consent (median = 3.5) in visiting health facilities.

Table 3 shows the comparison of the two groups after the intervention by Wilcoxson rank sum test. The *Mari Meter-X* score was reduced more in the intervention group than in the control group (median: − 5 and 0, respectively, *p* < 0.001). The STAI-Y state anxiety was lower in the intervention group than in the control group (median: − 3 and 0, respectively, *p* < 0.001). However, the face scale score did not differ between the two groups.

**Table 3:** Outcomes for intervention vs. control group by Wilcoxon rank sum test

	Intervention *n* = 119				Control *n* = 122				*p* value
		(Median)				(Median)			
	Q1	Q2	Q3	Range	Q1	Q2	Q3	Range	
Mari Meter-X	−15	− 5	0	(−54) − 18	-1	0	1	(−17) -42	0.001^***^
STAI-Y: state anxiety	− 11	−3	0	(− 37) -27	− 3	0	0	(−16) -20	0.001^***^
Face Scale	0	0	0	(−2) -4	0	0	0	(−3) -3	0.705

Q1 The first quartile

Q2 The second quartile

Q3 The third quartile

^***^p < 0.001

We found statistically significant differences between groups in countries of origin and experience of visiting medical facilities. However, the multiple regression analysis using STAI-Y scores showed that the intervention was still effective after adjusting for these factors. We found that no factors included in this study affected the effect of the intervention on the outcome.

## Discussion

This study found that the digital animation of the health system in Japan reduced concerns related to healthy living and illness prevention (measured with *Mari Meter-X*) and the intensity of feelings of anxiety (measured with STAI-Y). However, we did not see significant change in their feelings expressed using the face scale. Our results are similar to those in a previous study using digital animation in another health field, examining behavioral change in handwashing with soap to reduce diarrhea and respiratory infections [33]. We also found that the majority of international visitors did not obtain information regarding the Japanese health system before coming to Japan.

The main finding shows that viewing a comprehensive, yet succinct digital animation provides visitors with knowledge on how to access medical care or contact emergency services. Digital animation is more effective as it summarizes the most basic information to access health services in Japan. Presenting information with voice and animation makes it easier for the audience to understand the points in a short time than reading printed texts. Having prior knowledge of the health system and how to access medical care may reduce their anxiety [38]. The reason why face scale scores did not differ between the two groups after the intervention may be that the visitors started thinking about health care more seriously, realizing the risks after watching *Mari Info Japan* even though it was entertaining.

One possible explanation for the large proportion of international visitors, who did not obtain information regarding the Japanese health system before coming to Japan, is that they assume a developed country such as Japan has a similar public health system as in their home country. Although the information might have been available, the visitors did not access the latest health guidelines before travelling [38].

This study has several strengths. First, this study is the first to rate the effectiveness of a unique digital animation of Japanese health information on the anxiety experienced by international visitors. Second, the baseline demographic factors of participants were well-balanced between the two groups. The only exception was that participants who had visited medical facilities in Japan were more frequent in the control group, while those who were from China, Germany, Indonesia, New Caledonia, and Singapore were only in the intervention group. Third, sampling contamination was unlikely as we conducted the research with intervention and control groups on different days. Lastly, this study has a comparatively high participation rate of 88% (265 out of 300). Targeting those who were leaving Japan and waiting for their flights at the gates might have helped it as they had time to participate in this study.

This study also has some limitations. First, we could not ensure the participants were blind to their group assignment due to the nature of the interventions. However, there was very little chance that they viewed the other intervention and knew that they were allocated to a different intervention. In addition to the participants, the researchers who conducted the data collection and data analyses were not blinded to the group allocation. Second, we did not randomly assign participants to groups, which means that although we adjusted the effect of the intervention for other factors that could affect the outcome, there is still a chance that unknown factors, such as having friends in Japan and duration of their stay in Japan, might have affected the results. We could not examine if these factors were balanced between the intervention and control groups.

### Implications and future study

Our results revealed the advantages of distributing health information prior to visiting countries through simple and comprehensive methods. Additionally, we need to continuously update the content of health information based on recent events and provide a more effective dissemination of health information to international visitors prior to their arrival in Japan. Examples include web-based digital quizzes, information on multi-lingual TV channels popular among international visitors, and smart-phone applications. These tools and procedures to improve educational and communication structures will facilitate health care for international visitors to Japan. Future study on ways to provide this kind of information to international visitors on their arrival or during their stay at different places is required. A randomized controlled trial (RCT) will provide stronger evidence for the effectiveness of the intervention while avoiding unknown confounding factors.

## Conclusions

This study found that watching a digital animation is more effective in reducing anxiety among international visitors to Japan compared with reading a standard brochure because it presents the summary of important information in an enjoyable but succinct way. Introducing similar digital animations of health information as a main tool to provide necessary information to international visitors on their arrival may contribute to reducing their anxiety and avoiding troubles associated with health clinic visits.

## Additional files

Additional file 1: Contents of the information for the intervention group. The title is *Mari Info Japan*. It shows the headings for the digital animation for this study. (DOC 42 kb)

Additional file 3: Contents of information for the control group. It shows a simple information sheet in English for this study. (DOC 42 kb)

Additional file 4: *Mari Meter-X* questionnaire which examines the level of anxiety regarding visiting health facilities in Japan. *Mari Meter-X* questionnaire used to obtain primary data. It shows the full version of the questionnaire for this study. (DOC 51 kb)

## Abbreviations

NRS: Non-randomized controlled study
RCT: Randomized controlled trial
STAI-Y: The State-Trait Anxiety Inventory Form-Y
