# Supplementary material for: Providing Japanese health care information for international visitors: digital animation intervention
Source: BMC Health Serv Res. 2018 May 21;18:373. doi: 10.1186/s12913-018-3191-x (PMC5963085; doi:10.1186/s12913-018-3191-x)
Supplement: Supplementary file 2 — Contents of the information for the intervention group. The title is Mari Info Japan. It shows the headings for the digital animation for this study. (DOC 42 kb) [file 12913_2018_3191_MOESM1_ESM.doc]

Additional file 1. Contents of the information for the intervention group (headings for the digital animation)

**Mari Info Japan (Digital Animation)**

Japan is known as one of the world’s developed countries and maintains a constant standard for healthcare and nursing. There is no need to be concerned about water borne infectious diseases because of the high level of hygiene. You can easily find hospitals and clinics everywhere, apart from certain small islands. However, for foreigners who find themselves in unfamiliar towns, it will not be easy to visit hospitals and there could be problems for hospitals who receive them. This can be difficult sometimes because we do not know each other. Generally, information is written only in Japanese. Japan rarely diffuses information about health care and nursing to the rest of the world in a language other than Japanese. In order to make your medical visit easier, this guide will help you to receive healthcare and nursing in Japan.

Guide to medical visits and nursing in Japan

1. Health promotion and prevention, ②Payment and insurance for medical care, ③Language and communication, ④Informed consent, ⑤Selection of hospitals, ⑥Countermeasures for infectious diseases, ⑦Quality of nursing and medical care, ⑧Cultural background, ⑨Signs in hospitals and clinics, ⑩Relations with medical staff, ⑪Emergency medical care.

Information as of January 1, 2014, subject to modification
