# Supplementary material for: Providing Japanese health care information for international visitors: digital animation intervention
Source: BMC Health Serv Res. 2018 May 21;18:373. doi: 10.1186/s12913-018-3191-x (PMC5963085; doi:10.1186/s12913-018-3191-x)
Supplement: Supplementary file 3 — Contents of information for the control group. It shows a simple information sheet in English for this study. (DOC 42 kb) [file 12913_2018_3191_MOESM3_ESM.doc]

Additional file 3. Contents of information for the control group

**Medical care in Japan**

While the Japanese medical system is extensive and comprehensive, the level of care varies. Here are some things to note if you need to seek medical attention:

- It is better to seek care at university hospitals or other large hospitals rather than clinics.
- Japanese doctors and hospitals are sometimes reluctant to treat foreigners. It helps to carry proof of insurance and to be willing to show it. If a doctor or hospital seems reticent about giving care, you should insist on it (even though Japan has no Hippocratic oath, doctors can be told that they have to treat patients in need of care).
- Most hospitals and clinics have regular hours (usually in the mornings) when they will see patients.
- Hotels and *ryokan* that cater to foreigners will usually know the best hospitals in a particular area and will also know hospitals with English-speaking doctors.

Reference: Rowthorn C, et al. (2011). Japan-Lonely Planet Travel Guide, Lonely Planet, Melbourne, Australia
