# Supplementary material for: Providing Japanese health care information for international visitors: digital animation intervention
Source: BMC Health Serv Res. 2018 May 21;18:373. doi: 10.1186/s12913-018-3191-x (PMC5963085; doi:10.1186/s12913-018-3191-x)
Supplement: Supplementary file 4 — Mari Meter-X questionnaire which examines the level of anxiety regarding visiting health facilities in Japan. Mari Meter-X questionnaire used to obtain primary data. It shows the full version of the questionnaire for this study. (DOC 51 kb) [file 12913_2018_3191_MOESM4_ESM.doc]

Additional file 4. *Mari Meter-X* questionnaire

Below are some issues that concern some but not all visitors. Please read each statement and CIRCLE the response that best suits your imagination/experience. There is no right or wrong answer.

When you consult a medical facility in Japan, would you be concerned about following:

|  | | Not at all | Not very |  | Somewhat | Very |
| --- | --- | --- | --- | --- | --- | --- |
|  | | concerned | concerned | Neutral | concerned | concerned |
| 1 | Health Promotion | 1 | 2 | 3 | 4 | 5 |
| 2 | Pay Medical Expenses | 1 | 2 | 3 | 4 | 5 |
| 3 | Medical System | 1 | 2 | 3 | 4 | 5 |
| 4 | Language Communication | 1 | 2 | 3 | 4 | 5 |
| 5 | Informed Consent | 1 | 2 | 3 | 4 | 5 |
| 6 | Choose a Hospital | 1 | 2 | 3 | 4 | 5 |
| 7 | Pick up an Infection | 1 | 2 | 3 | 4 | 5 |
| 8 | Quality of Nursing Care | 1 | 2 | 3 | 4 | 5 |
| 9 | Quality of the Medicine | 1 | 2 | 3 | 4 | 5 |
| 10 | Life Style Difference | 1 | 2 | 3 | 4 | 5 |
| 11 | Eye Contact | 1 | 2 | 3 | 4 | 5 |
| 12 | Protection of Privacy | 1 | 2 | 3 | 4 | 5 |
| 13 | Directions in a Hospital | 1 | 2 | 3 | 4 | 5 |
| 14 | Dealing with Medical Staff | 1 | 2 | 3 | 4 | 5 |
| 15 | Emergency Care | 1 | 2 | 3 | 4 | 5 |
